# Dietary calcium intake and food sources among Chinese adults in CNTCS

**DOI:** 10.1371/journal.pone.0205045

**Published:** 2018-10-01

**Authors:** Feifei Huang, Zhihong Wang, Jiguo Zhang, Wenwen Du, Chang Su, Hongru Jiang, Xiaofang Jia, Yifei Ouyang, Yun Wang, Li Li, Bing Zhang, Huijun Wang

**Affiliations:** National Institute for Nutrition and Health, Chinese Center for Disease Control and Prevention, Beijing, China; Shanghai Diabetes Institute, CHINA

## Abstract

**Background:**

Calcium is one of the essential micronutrients in the human body and is well-known for its important role in keeping bones and teeth healthy. However, calcium deficiency is a very common nutritional problem in the world and especially in China. The aim of this research was to determine the dietary calcium intake of Chinese adults and the corresponding food sources based on data from the 2015 China Nutritional Transition Cohort Study.

**Methods:**

We obtained dietary data from Chinese adults ages 18 to 64 years in 15 provinces, autonomous regions, and municipalities using 3 consecutive days combined with the household weighing method. We used the *China Food Composition* (book 1, 2^nd^ edition) to calculate the calcium intake from each food category. We regarded the percentage of participants with a calcium intake median below the estimated average requirement as the level of calcium inadequacy in the overall population.

**Results:**

We divided the participants into 2 age groups, 18–49 years and 50–64 years, which included 6,630 and 5,307 participants, respectively. The groups’ dietary calcium intake medians were 324.8 milligrams per day (mg/d) and 332.7 mg/d, respectively, and the calcium inadequacies were 92.9% and 96.0%, correspondingly. The median calcium intake for the whole study population was 328.3 mg/d, and the inadequacy was 94.3%, which improved with higher education, income, and urbanization levels. The main food sources of dietary calcium among the study population were vegetables, legumes, and cereals, which contributed 30.2%, 16.7%, and 14.6%, respectively. Milk and dairy products contributed 6.7% to the study population but varied with the urbanization level to 15.0%, 6.1%, 6.3%, and 2.0% in urban, suburban, county, and rural areas, respectively.

**Conclusion:**

These findings highlight the importance of nutrition education and intervention for Chinese adults to improve their dietary structures and increase milk and dairy products intake to consume adequate calcium.

## Introduction

Calcium is one of the essential micronutrients in the human body and is well-known for its important role in keeping bones and teeth healthy [1]. However, calcium inadequacy is prevalent among adults in both developing and developed countries [2–6]. In Greece 18.2% to 59.3% of middle-aged women and 44.1% to 96.7% of postmenopausal women consumed calcium below the estimated average requirement (EAR) in 2008 [3]. In the United States the percentages were 6.2% for males and 26.2% for females ages 19–50 during 2007–2010 [2]. In the past we assessed the calcium inadequacy in Chinese adults by comparing the actual intake to the adequate intake (AI) level. Between 1991 and 2009 among adults 18–49 years old 94.8%–97.8% of males and 95.7%–98.6% of females showed intakes below the AI level [5]. Using the most recent Chinese Dietary Reference Intakes (DRI), published in 2013, we compared dietary calcium intakes to the EAR to determine the levels of inadequacy among different population groups [7]. Few studies report the national Chinese calcium intake. One study reported that during 2010–2012 the dietary calcium intake was 366.1 milligrams per day (mg/d) among Chinese age 2 and older [6]. But it did not report the specific intake of adults, and the data were out of date. Another study reported that in 2016 in 8 Chinese cities the median was 402.7 mg/d, excluding calcium supplements [8]. But since it only examined residents of cities, that study likely overestimated the dietary calcium intake, and its sample size was not large enough to be representative. Hence it was necessary to determine age-specific intakes in a larger Chinese population.

## Methods

### The China Nutritional Transition Cohort Study and study participants

We evaluated adults ages 18–64 who participated in the China Nutritional Transition Cohort Study (CNTCS), a national project financed by the National Institute for Nutrition and Health of the Chinese Center for Disease Control and Prevention. This project was a longitudinal follow-up study based on the China Health and Nutrition Survey was developed by the National Institute for Nutrition and Health and the University of North Carolina at Chapel Hill [9]. In 2015, 3 provinces (Shaanxi, Yunnan, and Zhejiang) were added to the original 12 areas. We drew samples from each of the provinces via a multistage, random cluster method. We excluded pregnant (number [n] = 51) or lactating (n = 103) women and adults reporting implausible energy intakes (< 500 or > 5,000 kilocalories per day, n = 61). We also excluded participants with dietary calcium intakes less than the 0.25 percentile or higher than the 99.75 percentile. Our sample totaled 11,937 participants. The institutional review board of the National Institute for Nutrition and Health approved the study protocol, ethics approval code 2015017 and approval date August 8, 2015. All subjects provided written informed consent prior to their participation in this research. We conducted the research in accordance with the Declaration of Helsinki.

### Dietary intake measures

Trained investigators obtained the dietary intake data in each province, autonomous region, and municipality. Using consecutive 3-day dietary recalls combined with the household weighing method, including 2 weekdays and 1 weekend day, investigators collected data on individual food consumption at home and away from home. With a household weighing method they compiled data on household oil and condiment consumption and calculated individual consumption according to the proportion of food each household member ate at home. The study required that the participants write down what they ate as soon as possible afterward to reduce recall bias. In addition a tablet device (Lenovo ThinkPad Tablet 2, China) showed the weights of a large number of foods as a reference for the participants.

### Calcium intake sources and assessment

We used the Chinese Food Composition Table to compute the calcium intake from each food the participants consumed [10]. We grouped foods not included in the table into the most similar categories. We expressed the total calcium intake for each person as the daily intake level (mg/d). We used the EAR cut-point method to estimate the population prevalence of calcium intake inadequacy. Applying this method both in China and abroad, we computed the population prevalence of inadequate intake as the proportion of the group with calcium intakes below the EAR [7, 11–14]. Because the EARs for calcium intake vary according to age, we show parts of our results separately for the 18–49 age group and the 50–64 age group [7].

### Statistical analysis

We expressed continuous variables as means ± standard deviations (SD), medians, 25^th^ percentile (P25), and 75^th^ percentile (P75) and categorical variables as percentages. With the Wilcoxon rank sum test, a nonparametric test, we compared the mean values of calcium intake between the 2 age groups. We used another nonparametric test, the Kruskal-Wallis rank sum test, to compare the mean values of calcium intake among more than 2 groups. For the meaningful multigroup results, we used the student-Newman-Keuls (SNK) test to find the differences between pairings of individual groups. A chi-square test compared the percentage of calcium intake below the EAR in areas with different urbanization levels. All p values reported are two-tailed. The level of statistical significance was p < 0.05. We conducted our statistical analysis with SPSS 21.0 and SAS 9.4.

## Results

### Characteristics of the study participants

Table 1 summarizes the characteristics of the 11,937 Chinese adults ages 18–64 included in this study. Of them 6,630 (55.5%) were 18–49 years old, 5,307 (44.5%) were 50–64 years old, and 52.9% were female.

**Table 1:** Sample size and dietary calcium intake of Chinese aged 18–64 years in 15 provinces (autonomous regions and municipalities) in 2015 (mg/d).

	18–49 years old						50–64 years old						18–64 years old					
	n (%)	Mean±SD	P25	Median	P75	*P*	n (%)	Mean±SD	P25	Median	P75	*P*	n (%)	Mean±SD	P25	Median	P75	*P*
Gender																		
Male	3102(46.8)	378.0±187.2	249.7	341.3	465.4	*<0*.0001^*^	2520(47.5)	394.5±202.5	251.6	354.8	481.5	*<0*.0001^*^	5622 (47.1)	385.4±194.3	250.4	347.2	469.7	*<0*.0001^*^
Female	3528(53.2)	348.9±188.7	222.1	311.0	423.4		2787(52.5)	360.5±199.3	225.7	315.2	442.1		6315 (52.9)	354.0±193.5	224.1	312.7	430.6	
Education																		
Primary school or below	1160(17.5)	335.4±186.7	212.3	293.2 ^a^	402.2	*<0*.*0001*^*†*^	1860(35.1)	341.6±181.1	219.2	304.1 ^a^	419.8	*<0*.*0001*^*†*^	3020 (25.3)	339.2±183.3	216.8	299.3 ^a^	412.7	*<0*.*0001*^*†*^
Middle school	2381(35.9)	350.0±176.2	230.6	315.2 ^b^	428.6		1764(33.2)	375.0±204.1	237.4	328.6 ^b^	456.2		4145 (34.7)	360.6±189.0	233.5	320.0 ^b^	439.6	
High school	1532(23.1)	363.6±184.4	240.6	328.7 ^c^	439.8		1344(25.3)	413.0±211.2	268.3	372.4 ^c^	509.7		2876 (24.1)	386.7±198.9	252.0	346.3 ^c^	469.5	
University or over	1557(23.5)	400.8±205.5	256.1	360.3 ^d^	496.1		339(6.4)	433.1±217.3	279.9	372.3 ^c^	530.6		1896 (15.9)	406.6±208.0	261.4	362.9 ^d^	500.5	
Region																		
Urban	1294(19.5)	427.4±214.1	282.5	386.0 ^a^	526.5	*<0*.*0001*^*†*^	1234(23.3)	437.0±225.2	277.5	385.9 ^a^	538.8	*<0*.*0001*^*†*^	2528 (21.2)	432.1±219.6	280.3	386.0 ^a^	530.2	*<0*.*0001*^*†*^
Suburb	1146(17.3)	370.6±180.5	247.1	334.6 ^b^	455.0		854(16.1)	386.0±190.9	256.1	347.8 ^b^	461.8		2000 (16.8)	377.2±185.1	250.2	339.9 ^b^	456.7	
County	1286(19.4)	346.9±170.5	230.6	319.7 ^c^	422.2		874(16.5)	361.2±193.1	235.5	319.1 ^c^	448.5		2160 (18.1)	352.7±180.1	232.9	319.3 ^c^	429.4	
Rural	2904(43.8)	337.3±180.0	217.9	300.3 ^d^	409.6		2345(44.2)	347.2±187.5	220.1	307.5 ^d^	425.4		5249 (44.0)	341.7±183.4	219.1	303.3 ^d^	416.5	
Household per-capita annual income																		
Low	2140(32.3)	355.0±192.2	223.6	315.3 ^a^	430.6	*<0*.*0001*^*†*^	1839(34.7)	362.7±204.5	225.2	320.0 ^a^	443.6	*<0*.*0001*^*†*^	3979 (33.3)	358.6±198.0	224.7	316.9 ^a^	436.7	*<0*.*0001*^*†*^
Middle	2217(33.4)	349.7±181.4	232.7	313.2 ^a^	424.4		1762(33.2)	364.1±187.3	236.0	320.1 ^a^	446.2		3979 (33.3)	356.1±184.2	234.4	316.8 ^a^	433.0	
High	2273(34.3)	382.1±190.4	249.9	346.4 ^b^	472.8		1706(32.2)	404.6±209.4	256.8	360.0 ^b^	500.6		3979 (33.3)	391.7±199.0	252.8	350.9 ^b^	483.2	
Total	6630(100.0)	362.5±188.5	234.8	324.8	443.0		5307(100.0)	376.6±201.5	238.6	332.7	461.0		11937 (100.0)	368.8±194.5	236.4	328.3	450.7	

^*^: Wilcoxon rank sum test of dietary calcium intake

^†^: Kruskal_Wallis rank sum test of dietary calcium intake

^a,b,c,d^: results of SNK(student-Newman-Keuls) test; different letters indicate significant differences between groups.

### Dietary calcium intakes of the study participants

Table 1 also summarizes the mean, the median, and some selected percentiles of participants’ dietary calcium intakes. The intakes were 328.3 mg/d, 324.8 mg/d, and 332.7 mg/d among the study population as a whole, the 18–49 age group, and the 50–64 age group, respectively. Intake among males was significantly higher than that among females, 347.2 mg/d compared to 312.7 mg/d. The higher the education level was, the higher the dietary calcium intake was. Participants with primary school or lower educations had the lowest dietary calcium intake at 299.3 mg/d, while those with university or higher educations consumed the highest at 362.9 mg/d. Similarly, intake increased with urbanization level. The intake was 386.0 mg/d for urb, 339.9 mg/d for suburb, 319.3 mg/d for county, and 303.3 mg/d for rural areas. They were all significantly different. In addition the intake increased with the household per capita annual income. Participants with high household incomes consumed about 34.0 mg/d more than those with low or middle incomes. These trends also appeared in both age groups.

### Assessment of dietary calcium intakes of the study participants

As Table 2 shows, inadequate dietary calcium intakes were widespread among our study participants. In the study population as a whole dietary calcium intake below the EAR was 94.3%. It was significantly lower among those 18–49 years old at 92.9% than those 50–64 years old at 96.0%. Specifically, regardless of region of residence, more participants in the 50–64 age group, 93.0%–97.3%, than in the 18–49 age group, 87.8%–94.3%, did not meet the dietary calcium EAR. The percentage of dietary calcium intake below the EAR was 90.4% in urban areas, which was significantly lower than that in suburban, county, and rural areas at 94.8%, 95.4%, and 95.5%, respectively.

**Table 2:** Percentages of age and regions-specific adults with intakes below EAR for calcium.

Age groups	Regions				
	Urban	Suburb	County	Rural	Total
18–49 years old^*^	87.8	93.6	94.1	94.3	92.9
50–64 years old^*^	93.0	96.4	97.3	97.0	96.0
18–64 years old^*^	90.4	94.8	95.4	95.5	94.3

^*^: *P*<0.001

### Food sources of dietary calcium

Table 3 reveals several food groups’ percentages of contributions to dietary calcium intake. Vegetables were the major sources in all populations, accounting for 30.2%, legumes followed at 16.7% and then cereals at 14.6%. This trend also appeared in both age groups. Other food groups, including fish, shrimp, and shellfish; milk and dairy products (liquid milk, powder, yoghurt, cheese, etc. Beverage with milk are not included.); condiments; snacks and fast foods; eggs; and meats, contributed less than 10.0% in suburb, county, and rural areas. Among all age groups vegetables were the major sources of calcium in urban areas, followed by milk and dairy products, which contributed 15.0–15.1%. This was significantly higher than in suburban and county areas, where it contributed 5.2–6.9%, and rural areas, where it contributed 2.0%.

**Table 3:** Percent contribution of main food groups in the total dietary calcium intake by age-specific groups in China (%).

Food groups	18–49 years old				50–64 years old				18–64 years old				
	Urban	Suburb	County	Rural	Urban	Suburb	County	Rural	Urban	Suburb	County	Rural	Total
Vegetables	23.7	32.6	28.8	32.8	24.4	34.9	30.1	32.9	24.0	33.6	29.3	32.9	30.2
Legumes	13.8	16.9	16.2	18.1	14.1	17.5	16.0	18.9	13.9	17.2	16.1	18.5	16.7
Cereals	10.8	12.7	15.4	17.9	10.3	12.2	15.1	17.3	10.6	12.5	15.3	17.6	14.6
Fish, shrimp, shellfish	9.3	6.6	7.4	5.6	9.5	6.8	8.1	6.1	9.4	6.7	7.7	5.8	7.2
Milk and dairy products	15.1	6.9	6.6	2.0	15.0	5.2	5.8	2.0	15.0	6.1	6.3	2.0	6.7
Condiments	4.8	4.3	4.6	5.3	4.6	4.2	4.6	6.0	4.7	4.3	4.6	5.6	5.0
Snack, fast foods	6.9	3.9	4.8	3.6	6.5	3.9	5.4	3.2	6.7	3.9	5.0	3.4	4.6
Eggs	3.7	3.2	4.6	4.2	3.9	3.4	4.2	3.9	3.8	3.3	4.4	4.1	3.9
Meats	3.5	3.7	3.5	2.9	2.8	3.1	2.9	2.4	3.1	3.4	3.3	2.7	3.0

## Discussion

In the past researchers have assessed populations’ calcium intake levels by comparing actual intake to AI values [5]. With the publication of the Chinese DRI in 2013, EAR replaced AI in assessing inadequacy. However, few studies assessed calcium intake inadequacy through EAR. Huijun Wang examined the usual daily micronutrient intake of Chinese children in 2017 [15]. The China National Nutrition and Health Survey in 2002 reported dietary calcium intake among Chinese adults [16], and another surveyed Chinese adults ages 18 to 75 in 8 cities [8]. But either the data were out of date or the sample size was not large enough in these studies. Our study is the first to estimate the most recent dietary calcium intake inadequacies among Chinese adults with the EAR cut-point method and a comparatively enormous sample size.

The results indicate that dietary calcium intake was severely inadequate in China even though the dietary structure has changed in recent decades. The Chinese EAR for dietary calcium intake is 650.0 mg/d for adults ages 18–49 and 800.0 mg/d for those ages 50–64 [7]. However, our study found that intake was 324.8 mg/d for those 18–49 and 332.7 mg/d for those 50–64, essentially half the EAR levels.

The dietary calcium intake of Chinese adults was not much different from that of adults in other Asian countries. For example, the medians were 292.0 mg/d for women and 306.0 mg/d for men 40 years old or over in South Korea [17], 522.0 mg/d–680.0 mg/d for adults 40–59 years old in Japan [18], and 295.0 mg/d–619.0 mg/d for Chinese adults 45–74 years old in Singapore [19]. These figures are significantly lower than those in some developed countries. The dietary calcium intakes were 933.0–1,025.0 mg/d for adults 19–70 years old in the United States [2], 770.0–934.4 mg/d for those 18–64 in Spain [20], and 833.6–1,013.6 mg/d for those 18–79 in France [21].

The disparity may be due to the diversity of the dietary structures in different countries. A large portion of the Chinese population may not meet the calcium DRI because of poor dietary choices. Between 1991 and 2009 among Chinese adults vegetables were the leading food source of dietary calcium, contributing 28.0–45.3%, followed by cereals and legumes sequentially [5]. Nowadays, vegetables was still the leading source of dietary calcium, contributing 30.2%. However, legumes, at 16.7%, had replaced cereals as the second highest contributor. These 3 food groups accounted for 61.5% of the total dietary calcium intake. In fact, milk and dairy products were the optimum food source of dietary calcium which made nearly half contribution to the U.S. and Spanish population’s calcium intake [22, 23]. In contrast, Chinese adults consumed 21.3 grams of milk and dairy products per day, 6.7% of the total dietary calcium intake [24]. The contributions were almost the same in both of our age groups, but it increased with the urbanization levels. In 2009 milk and dairy products contributed 8.3%, 3.9%, 2.8%, and 0.8% of dietary calcium in urban, suburban, county, and rural areas, respectively. In 2015 the contributions increased to 15.0%, 6.1%, 6.3%, and 2.0%, respectively [5]. These contribution percentages are in line with Chinese adults’ milk and dairy product consumption in 2015 [24]. T. A. Nicklas et al. have shown that consumption of more dairy can reduce the prevalence of calcium deficiency [25]. Therefore it is necessary for the Chinese to increase consumption of milk and dairy products to reduce calcium inadequacies, especially in areas with low urbanization levels.

A limitation of our research is that we did not examine the use of calcium supplements, which likely had a small effect on the calcium intake calculations. One study indicated that the rate of calcium supplement use among Chinese adults was 10.6% and that the calcium intake among adults taking supplements was 25.8 mg/d higher than that among those not taking supplements, which was negligible in 2016 [8]. In addition it is still controversial whether or not calcium supplements are associated with an excess risk of cardiovascular disease [26–29]. Therefore we do not recommend elevating calcium intake with supplements.

In conclusion, the dietary data in the 2015 CNTCS show that the dietary calcium intake among Chinese adults was severely insufficient and well below the calcium DRI. Vegetables, legumes, and cereals are the main sources of dietary calcium among this population. It is urgent that China improve the population’s poor dietary choices and promote consumption of milk and dairy products.
